# Supplementary material for: Factors Associated with Vaccination Adequacy in People Living with HIV: A Cross-Sectional Study
Source: Vaccines (Basel). 2024 Sep 1;12(9):1003. doi: 10.3390/vaccines12091003 (PMC11435921; doi:10.3390/vaccines12091003)
Supplement: Supplementary file 1 [file vaccines-12-01003-s001.zip › vaccines-3156865-supplementary.pdf]

Table S1. Fit indices for model for Hosmer-Lemshow goodness fit statistics.

Model	X^2	df	*p*-value
Double Adult	18.2439	13	0.1485
Hepatitis B	5.7218	13	0.9557
Hepatitis A	11.4485	13	0.5733
HPV	12.0177	13	0.5262
13-valent pneumococcal	6.6939	13	0.9172
23-valent pneumococcal	11.4255	13	0.5752
Meningococcal C	6.7396	13	0.9151
Measles/Mumps/Rubella	10.1338	13	0.6830
Yellow Fever	10.7265	13	0.6337
Adequate Scheme	5.7055	13	0.9562

$\chi2$: ratio Chi square; df: degrees of freedom ratio
